# Assessing Maternal Satisfaction: Patient-Centered Care, Hospital Environment, and Information-Seeking in Chanika Hospital in Tanzania

**DOI:** 10.3390/jpm14050455

**Published:** 2024-04-25

**Authors:** Sarang Jang, Sangmi Lee, Aeree Sohn

**Affiliations:** 1 Department of Public Health, Sahmyook University, Seoul 01795, Republic of Korea; srjang@syu.ac.kr; 2 Medipeace, Seoul 08390, Republic of Korea; sangmi.lee@medipeace.org

**Keywords:** maternal satisfaction, patient-centered care, hospital environment, information-seeking

## Abstract

This study examined the impact of patient-centered care, satisfaction with the hospital environment, and maternal information-seeking on maternal healthcare satisfaction in Tanzania. A total of 707 mothers who delivered at Chanika Hospital in Tanzania were surveyed using a structured questionnaire. Multiple regression analyses were conducted to identify factors related to maternal satisfaction. Only 9.9% of the participants reported that they “usually” or “always” felt involved in treatment decisions. High levels of satisfaction were found for hospital cleanliness (93.6%) and safety (94.9%). However, there was a significant gap in satisfaction regarding the adequacy of water quality for medical services, with only 8.1% expressing satisfaction. Limited use of digital platforms was observed in terms of information-seeking behavior for fetal development, with only 19.5% of the participants using the internet and 14.3% using mobile apps. Patient-centered experiences with healthcare providers, especially midwives, had a significant positive impact on maternal satisfaction (β = 0.11, *p* = 0.021). Other significant variables were satisfaction with the hospital environment (β = 0.25, *p* < 0.001) and satisfaction with hospital water (β = 0.13, *p* < 0.001). It is recommended that healthcare improvements focus on patient-centered experiences and water quality for drinking and medical services to improve patient satisfaction.

## 1. Introduction

Tanzania faces a critical maternal health challenge characterized by a high maternal mortality ratio of 238 per 100,000 live births in 2020, well above the 135 per 100,000 live births reported for Zambia [1]. Several interrelated factors have influenced this situation. Delayed decision-making in seeking maternal healthcare is a major barrier, often due to a lack of awareness among pregnant women and their families about the signs of childbirth and labor [2,3]. This issue is further complicated by financial constraints and limited autonomy in choosing the place of delivery, which are common challenges in low-resource settings [4].

In addition, logistical barriers, such as poor road conditions and unreliable transportation, significantly hinder access to health facilities [3]. Once at a health facility, women often face challenges such as a lack of trained medical staff, insufficient medical supplies, and inadequate infrastructure, which negatively impact the quality of care they receive [4,5].

Healthcare disparities are common in developing countries, particularly in terms of maternal and child health [3,4,5]. Patient-centered care, which emphasizes patients’ needs and preferences, has been shown to improve health outcomes, reduce healthcare costs, and increase patient satisfaction [6]. In the context of maternal and child health, patient-centered care fosters better patient–clinician relationships, promotes shared healthcare decision-making, and enhances self-management skills. Studies have demonstrated the direct and indirect effects of patient-centered care on the use of prenatal and postnatal care services.

Patient perceptions and satisfaction are influential factors in future health-seeking behavior and are closely related to levels of trust in health systems [7,8]. Ethical perspectives emphasize the importance of adopting patient-centered care, which is consistent with health professionals’ duty to prioritize patient well-being and respect patient autonomy [7,8]. In particular, patient satisfaction is an important measure of patients’ experiences and outcomes when receiving healthcare services [7]. It serves as an important indicator of how well healthcare systems and providers are meeting patients’ needs and expectations and improving the patient experience [9].

Previous studies have highlighted the role of patient satisfaction in influencing antenatal care, maternal health-seeking behavior, and treatment adherence in Africa [10]. Satisfied patients are more likely to seek care again and recommend it to others [11]. Ensuring access to specialized health services, such as facility-based delivery, antenatal care, and postnatal care, is critical to reducing maternal mortality in Africa [12]. A study on patient satisfaction among pregnant women in Tanzania, Kenya, and Malawi found that factors such as women’s education level, parity, health provider education, and waiting time for services influenced satisfaction [13].

Therefore, this study examined the effects of various factors on maternal patient satisfaction with obstetric care in Tanzania. The study focused on communication experiences with different types of healthcare provider, treatment process, hospital environment, hospital water quality, and patient characteristics. The aim was to explore several dimensions of maternal healthcare, including patient-centered experiences, fairness of treatment, satisfaction with the hospital environment, and health information-seeking behavior related to fetal development, with special emphasis on antenatal care (ANC) and postnatal care (PNC) at Chanika Hospital in Tanzania.

## 2. Materials and Methods

### 2.1. Study Design

This study was conducted as part of a project in Tanzania supported by the Korea International Cooperation Agency (KOICA) under the Official Development Assistance (ODA) framework. The research team participated in this study as a component of Medipeace’s capacity-building program at Chanika Hospital in Tanzania.

Using a cross-sectional study design, this study used computer-assisted personal interviewing (CAPI) for data collection. Ethical approval was obtained from the National Institute for Medical Research (NIMR Act No. 23 of 1979) in Tanzania.

### 2.2. Participants and Procedures

The study population consisted of women who delivered at Chanika Hospital, located in the Ilala district of Dar es Salaam, Tanzania. In 2021, the hospital had an average daily attendance of 300 new mothers and performed approximately 35 births. Women who delivered between June and July 2022 were eligible to participate in the study. In this study, sample size was calculated using the G*Power 3.1 program based on the significance level (0.05), moderate effect size (0.3), and power according to Cohen’s criterion (0.80), which are necessary for the *t*-test, one of the data analysis methods. The required sample size was determined to be 580. Considering a survey response rate of 70–90%, the sample size for this study was set to 710. Ultimately, 707 participants responded.

The questionnaire was developed in consultation with key stakeholders, particularly hospital staff, to facilitate effective planning and implementation. In addition, research assistants were trained in the study’s tools, methods, and protocols for contingencies such as unstable internet connectivity. They applied CAPI (computer-assisted personal interviewing), a face-to-face interview method, to respondents. Specifically, the researcher assistants provided the respondents with a tablet that linked to the local-language (Kiswahili) questionnaire and had the respondents answer the questionnaire themselves.

### 2.3. Measures

This study used a detailed 36-item questionnaire to assess different aspects of the maternal healthcare experience, drawing on the Organization for Economic Co-operation and Development Healthcare Quality Indicators of patient experience [14], the US Centers for Medicare & Medicaid Services Hospital Consumer Assessment of Healthcare Providers and Systems Survey [15], and the UK National Health Service Imperial Survey core questionnaire [16]. The questionnaire included 12 items on patient-centered experiences with nurses, midwives, and doctors; six questions on general care, including explanations of side effects and pain relief; one question on fair treatment and two questions on patient rights; six questions on satisfaction with the hospital environment; and a 10-point patient-centered satisfaction scale. Two questions focused on information-seeking behavior and six on demographic characteristics such as age, education, length of marriage, and first birth status.

Most items were responded to using a 4-point Likert scale (“never”, “sometimes”, “usually”, “always”), except for satisfaction with the hospital environment, which used a 5-point scale (“very dissatisfied” to “very satisfied”). For analysis, these scales were dichotomized, combining “never” and “sometimes” into one category and “usually” and “always” into another for the 4-point scale, and combining “very dissatisfied”, “dissatisfied”, and “neutral” into “unsatisfied” and “satisfied” and “very satisfied” into “satisfied” for the 5-point scale.

The independent variables included measures of patient-centered experience, overall care, fair treatment, patient rights, and satisfaction with the hospital environment. Health information-seeking behaviors, specifically the use of the web or mobile platforms, were also independent variables. The scale for each variable was the sum of item responses, with higher scores indicating more positive experiences or behaviors. The dependent variable was the patient-centered satisfaction score. Demographic characteristics were included as control variables in the analysis. In addition, this study examined differences in patient-centered experiences and satisfaction based on the receipt of antenatal care (ANC) and postnatal care (PNC). ANC was defined as women receiving at least four antenatal visits and PNC as women receiving at least two postnatal visits within 48 h of delivery.

### 2.4. Data Analysis

In this study, data analysis was conducted utilizing IBM SPSS Statistics 26 software, with descriptive statistics, chi-square test, binomial logistic regression, and stepwise multiple regression models employed. Descriptive statistics presented the percentage distributions of respondents’ demographic characteristics, compared between women with satisfied and unsatisfied utilization of ANC and of PNC, and examined with the chi-square test. Patient-centered experiences in interaction and communication with healthcare providers were examined separately for nurses, midwifes and physicians. We then examined the patient-centered experiences in general care, fairness, and health information-seeking behavior for fetal development, and hospital environmental satisfaction compared between utilization status for ANC and for PNC. The logistic regression model was used to estimate the odds ratio (OR) and 95% confidence interval (CI) associated with the satisfaction. We also determined if there were differences in patient satisfaction with the hospital environments based on the utilization of ANC and of PNC. We further used the non-parametric Mann–Whitney test to examined the patient-centered satisfaction scores by ANC status and PNC status. Finally, the stepwise multiple regression model was employed to identify factors associated with patient satisfaction. The multicollinearity test among independent variables in the multiple regression analysis revealed that the variance inflation factor (VIF) values for all variables were below the threshold of 10, indicating no multicollinearity issues.

## 3. Results

### 3.1. Participants’ Characteristics

With a mean age of 27.4 (SD 5.9) years, 60.0% of participants were aged in their 20s among the 707 eligible women in the data analytics (Table 1). Regarding ANC, 352 participants (49.8%) had fewer than four visits, while 355 (50.2%) had four or more visits for adequate ANC compliance. Women with more than elementary education were more likely to have adequate ANC visits than women with less education (*p* = 0.042). Distributions of age, marriage duration, and number of times giving birth were not different between women with and without adequate ANC. On the other hand, 626 participants (90.6%) received adequate PNC visits within the first two days after delivery, whereas 65 participants did not.

### 3.2. Patient-Centered Experiences by Health Providers

In examining maternal experiences in interaction with healthcare providers, distinct patterns emerged across various domains, such as respect and courtesy, listening effectiveness, clarity in explaining hospital procedures, and responsiveness to requests. Table 2 shows that, in general, patient–provider interaction and communication activities were adequate in the maternal care for more than 60%. Midwives consistently demonstrated the most positive patient–provider interactions: 95.5% for respect and courtesy, surpassing nurses at 91.5% and doctors at 85.7%.

In contrast, doctors had the highest rate of negative patient–provider interactions across all assessed areas. Specifically, 14.3% of doctor interactions were rated as never or sometimes respectful and courteous, compared to 8.5% of nurses and 4.5% of midwives. In terms of responsiveness to requests, doctors showed a significant disparity, with a 60.4% negative interaction rate, which was much higher than that of nurses (36.6%) and midwives (34.9%).

### 3.3. Patient-Centered Experiences in General Care, Fair Treatment and Guarantee of Rights, Satisfaction with Hospital Environment, and Health Information-Seeking Behavior for Fetal Development

Most of the patients indicated they were always informed before given any medication, test, or treatment for ANC (74.3%) and PNC (75.8%) (Table 3). Women with adequate ANC visits were much more positive than women without adequate ANC (92.4% vs. 56.0%, OR = 9.6 (95% CI = 6.1–14.9), whereas the satisfaction in women with adequate PNC was not higher than in women without adequate PNC (74.0% vs.93.8%; OR= 0.2, 95% CI = 0.2–0.5). Similarly, adequate ANC users were more likely to be explained the side effects (83.1% vs. 27.0%; OR = 13.3, 95% CI = 9.2–19.1), whereas the corresponding rate was lower in women under PNC (8.3% vs. 27.7%, OR = 0.63, 95% CI = 0.4–1.1). However, a high portion of women indicated the failure of placing patient’s interests first, and were significantly less likely to practice ANC (54.4% vs. 88.9%; OR = 0.1, 95% CI = 0.1–0.2), whereas only 1.4% of women had the comment in the PNC period. Only 10% of women had involvement in the treatment decision. Our data also show that the study participants rarely could use internet search and mobile apps, particularly for women with inadequate ANC. In general, the satisfaction status in patients care varied between ANC and PNC.

### 3.4. Satisfaction with Hospital Environment

Table 4 shows that near 90% or higher portions of participants were satisfied with the hospital environment regarding cleanliness, safety and comfort, and water for handwashing, regardless of the ANC and PNC status. Women with adequate adherence to ANC and PNC were also more likely to report being satisfied with the hospital water quality, but less than 10% of all women were satisfied with the drinking water. Slightly over 60% of women were satisfied with the hospital laundry cleaning, which was greater in women with adequate ANC than in those without. However, a lower portion of women with adequate PNC were satisfied with the laundry condition. Satisfaction with water quality for medical service was lower in women with adequate ANC than in women without (23.7% vs. 54.0%; OR = 0.3, 95% CI = 0.2–0.4), whereas it was greater in women with adequate PNC than those without (40.4% vs. 7.7%; OR = 8.1, 95% CI = 3.2–20.5).

### 3.5. Patient-Centered Satisfaction Score and Factors Influencing Maternal Satisfaction Scores

Table 5 shows that the mean satisfaction score for all participants was 8.4 (SD = 1.5), based on a scale of 0–10; this was slightly higher in the non-ANC group than in the adequate ANC group and the difference was not significant. The mean maternal satisfaction scores of the adequate PNC and non-PNC groups were 8.4 (SD = 1.5) and 8.9 (SD = 1.4), respectively, and the difference was statistically significant with a U value of 16,072.00 (*p* = 0.004).

Multiple regression analysis further identified the factors influencing maternal satisfaction scores, which ranged from 0 to 10. Results show that demographic variables of age, marital status, and education level alone could not adequately explain the variance in maternal satisfaction scores (Table 6, Model 1). In contrast, the R-square value was improved to 0.110 (*p* = 0.021) in Model 2 with the inclusion of both demographic and hospital-related variables, confirming significant satisfaction in patient-centered experience with midwives (β = 0.11, *p* < 0.001), the hospital environment (β = 0.25, *p* < 0.001), and hospital water quality (β = 0.13, *p* < 0.001).

## 4. Discussion

Our study provides important insights into the healthcare experience, particularly in the context of maternal care. One of the most striking findings was the significant role played by patient-centered experiences with healthcare providers. Mother-centered care can lead to higher levels of mother satisfaction, better adherence to medical regimens, and improved health outcomes. The psychological benefits of mother-centered care include reduced anxiety and improved mental health.

Regarding patient-centered experiences in general care, we found that only 9.9% of the participants usually or always felt involved in treatment decisions. This is remarkably low compared with the reported national averages. It is important that women should give birth in settings in which they can participate in decision-making regarding their birth [17]. Shared counseling between patients and healthcare providers in decision-making about delivery options, such as cesarean section, has been reported to be effective in reducing decisional conflict and regret [17]. Despite the importance of involving patients in maternity care decisions, achieving this goal is not always easy. It can take a considerable amount of time for health professionals to involve patients in treatment decisions [18]. Facilitating patient participation in decision-making requires specific communication skills on the part of health professionals [19], and there are limitations in providing medical information about treatment to support patient participation [20]. In addition, some doctors do not want to be equals with patients in the decision-making process, and some patients want doctors to make treatment decisions for them [21].

In the areas of fair treatment and assurance of rights, our data showed that only 1.6% of the participants felt that they usually or always had the opportunity to make a complaint. A study comparing patient-centered experiences in general hospitals and hospitals in South Korea also found that the mean value of experiences related to ensuring patients’ rights was lower than in other areas of hospital experiences [22]. It is important that complaint reporting systems in healthcare institutions work from the perspective of patients rather than healthcare professionals [23]. Patient dissatisfaction is related not only to treatment outcomes, but also to concerns about treatment and lack of communication with healthcare providers [23]. Over a 26-month period in the United States, hospitalist complaints were 3.5 per 1000 hospitalizations, with the most common complaints related to communication with providers [24]. In an analysis of 200 complaints received over a five-year period about maternity services in Australia, the most common issues were rude behavior and poor communication [25]. To practice patient-centered care, patients must be informed that they can complain about hospital services at any time, feel comfortable doing so, and have hospital systems in place to rectify complaints as quickly as possible.

In this study, satisfaction with the hospital environment was high: 93.6% for cleanliness, and 94.9% for a safe and comfortable environment. The reason for high satisfaction with the hospital environment in developing countries may be that the quality of medical care is improving compared to the past, leading to improved satisfaction [26]. The general standard of care in hospitals may not be as high in developing countries; therefore, patients’ expectations of hospitals may be different from those in developed countries, which may explain higher satisfaction with the hospital environment in developing countries [27]. Although patients may be satisfied with the hospital environment for various reasons, patient satisfaction with the hospital environment varies across countries and hospitals. In a study on patient satisfaction in private and public hospitals in Bangladesh, 72.4% of patients reported they were satisfied with the cleanliness of hospital facilities, which was higher than their satisfaction with maintaining adequate privacy (65.4%) and communication with medical staff (42.4%) [27]. In contrast, in a Vietnamese study, satisfaction with the attitude and professionalism of medical staff was rated higher than 4 out of 5, whereas satisfaction with the hospital environment was rated lower than 4 [28].

Satisfaction rates dropped significantly when it came to specific aspects such as water quality for medical services, where only 8.1% expressed satisfaction. In hospitals, one’s expectations may be higher for drinking or therapeutic water than for general water use. A review of studies of satisfaction with water quality in hospitals showed that the results varied from country to country. In studies of inpatients in northern India and mothers in Ghana [29], approximately half reported having access to potable water. Studies that have examined the impact of water and sanitation in health facilities in low- and middle-income countries, as well as drinking water, on patient satisfaction have found that some mothers prefer home delivery to hospital delivery because of poor water quality in hospitals [30]. Water, sanitation, and hygiene (WASH) have been identified as key factors for improving patient satisfaction with maternity care, particularly in developing countries [30,31]. In developing countries, water quality is often poor, and mothers are less satisfied with the quality of water used for medical care, which should be selected as a priority at the national level.

In terms of health information-seeking behavior for fetal development, only 19.5% of the participants turned to the internet and 14.3% used mobile apps. Globally, the internet has become one of the most frequently used sources of information for pregnant women [32,33]). In a sample of pregnant women from 24 countries, more than 90% reported using the internet for pregnancy-related information [34]. The use of the internet and mobile phones to seek health information improves access to health information for pregnant women [33] and can be a useful tool for the self-management of health conditions, including personal experiences during pregnancy [35], safety of medication use during pregnancy [36], and healthcare information [35]. However, in developing countries, high illiteracy rates, poor information and communications technology infrastructure, and multiple responsibilities assigned to women have been shown to hinder health information-seeking behaviors, such as using the internet and mobile phones. However, the results of this study showed significantly lower levels of internet-based health information-seeking among ANC and PNC patients in Tanzania compared to a previous study [37]. Therefore, in developing countries, various efforts are needed to provide online and offline pregnancy-related information and improve digital health literacy at the community and hospital levels.

The regression models highlighted several factors that did not significantly influence maternal satisfaction, including age, marital status of three years or more, and education level. Although there is no consensus in the literature on the factors associated with satisfaction with antenatal care, several studies have found education, income level, and age are significantly associated with client satisfaction with antenatal care [30]. However, previous studies in Tanzania and Belgium found that lower levels of education and income were associated with significantly higher expectations of antenatal care but not with satisfaction with antenatal care [38]. Although we did not examine the mothers’ expectations in this study, we can cautiously speculate that socioeconomic level may influence satisfaction through expectations. As satisfaction with antenatal care is a complex phenomenon influenced by many factors, careful efforts are needed to design and implement management strategies that consider the diverse characteristics of mothers to ensure their continued access to antenatal and postnatal services.

The limitations of this research are as follows: Firstly, the survey utilized patient satisfaction evaluation criteria primarily employed in advanced nations, targeting mothers in Tanzania, which is a developing country. Although the investigation adhered to validated assessment criteria, further research necessitates the use of validated survey instruments tailored to the Tanzanian context and aimed at Tanzanian mothers. Moreover, as the study participants consisted of mothers delivering at a specific hospital in Tanzania, generalizing the findings to Tanzanian mothers at large presents a challenge. Therefore, future research endeavors should incorporate more meticulous designs, considering factors such as participants’ residency and hospital characteristics, to facilitate profound analysis.

## 5. Conclusions

Our study has several implications for healthcare policies and practice. Efforts should be directed toward improving patient-centered experiences, particularly in the area of shared decision-making. In addition, immediate interventions are needed to address specific areas of dissatisfaction, such as the quality of medical services. Overall, our findings provide a comprehensive understanding of the factors that influence maternal satisfaction and offer actionable insights for healthcare providers seeking to improve patient experience and satisfaction. The variables identified as significant predictors of maternal satisfaction serve as areas for healthcare providers to focus on for improvement. Given the complex web of factors that influence maternal satisfaction, it is clear that a multifaceted mother-centered strategy is essential for healthcare organizations seeking to improve the quality of their services.

## Figures and Tables

**Table 1:** Sociodemographic characteristics of participants with and without adequate ANC and PNC.

			ANC (*n* = 707)					PNC (*n* = 691)			
		Total (*n* = 707)	Yes (*n* = 355)	No (*n* = 352)	χ^2^	*p*	Total *(n* = 691)	Yes (*n* = 626)	No (*n* = 65)	χ^2^	*p*
		%	%	%				%	%		
Age (years)	10s	6.5	7.6	5.4	2.56	0.278	6.5	6.5	6.2	0.02	0.991
	20s	60.0	61.1	58.8			60.1	60.1	60.0		
	30s	33.5	31.3	35.8			33.4	33.4	33.8		
Marriage duration	Less than 3 years	50.1	52.7	47.4	1.94	0.176	50.1	50.3	47.7	0.16	0.698
	More than 3 years	49.9	47.3	52.6			499.9	49.7	52.3		
Education	Less than elementary	48.9	45.1	52.8	4.27	0.042	49.1	49.0	49.2	0.00	1.000
	More than elementary	51.1	54.9	47.2			50.9	51.0	50.8		
Times giving birth	First born	36.6	38.3	34.9	0.86	0.391	36.9	37.1	35.4	0.07	0.893
	Multiple births	63.4	61.7	65.1			63.1	62.9	64.6		

Sixteen participants were excluded who did not receive PNC within two days or responded that they did not know as of the survey date. ANC = antenatal care; PNC = postnatal care.

**Table 2:** Patient-centered experiences by health providers.

		Health Provider		
		Nurse	Midwife	Doctor
		%	%	%
Respect/courtesy	Never/sometimes	8.5	4.5	14.3
	Usually/always	91.5	95.5	85.7
Listening	Never/sometimes	9.1	4.4	13.0
	Usually/always	90.9	95.6	87.0
Explaining hospital procedures	Never/sometimes	23.3	16.4	29.7
	Usually/always	76.7	83.6	70.3
Handling requests	Never/sometimes	36.6	34.9	60.4
	Usually/always	63.4	65.1	39.6

**Table 3:** Patient-centered experiences in general care, fair treatment and guaranteeing of rights, patient experience of the hospital environment, and health information-seeking behavior for fetal development.

			ANC (*n* = 707)						PNC (*n* = 691)					
			Total (*n* = 707)	Yes	No	OR	95% CI	*p*	Total (*n* = 691)	Yes	No	OR	95% CI	*p*
			%	%	%				%	%	%			
			General Care											
Explanation before medication/ test/treatment	Never/sometimes	Ref.	25.7	7.6	44.0				24.2	26.0	6.2			
	Usually/always		74.3	92.4	56.0	9.6	6.1–14.9	<0.001	75.8	74.0	93.8	0.2	0.1–0.5	0.001
Explaining side effects	Never/sometimes	Ref.	44.8	16.9	73.0				89.9	91.7	72.3			
	Usually/always		55.2	83.1	27.0	13.3	9.2–19.1	<0.001	10.1	8.3	27.7	0.63	0.4–1.1	0.094
Involvement in treatment decision-making	Never/sometimes	Ref.	90.1	87.6	92.6				89.9	91.7	72.3			
	Usually/always		9.9	12.4	7.4	1.8	1.1–3.0	0.027	10.1	8.3	27.7	0.2	0.1–0.4	<0.001
Considering embarrassment during treatment	Never/sometimes	Ref.	5.8	6.8	4.8				5.9	5.9	6.2			
	Usually/always		94.2	93.2	95.2	0.7	0.4–1.3	0.274	94.1	94.1	93.8	1.0	0.4–3.0	0.937
Efforts to reduce pain	Never/sometimes	Ref.	31.0	20.3	41.8				29.4	30.7	16.9			
	Usually/always		69.0	79.7	58.2	2.8	2.0–4.0	<0.001	70.6	69.3	83.1	0.5	0.2–0.9	0.023
			Fair treatment and guaranteeing of rights											
Unfair treatment experience	Never/sometimes	Ref.	94.9	94.6	95.2				94.90	95.40	90.80			
	Usually/always		5.1	5.4	4.8	1.1	0.6–2.2	0.752	5.10	4.60	9.20	0.5	0.2–1.2	0.115
Possibility of filing complaints	Never/sometimes	Ref.	98.4	98.9	98.0				98.6	98.6	98.5			
	Usually/always		1.6	1,1	2.0	0.6	0.2–1.9	0.361	1.4	1.4	1.5	0.9	0.2–7.5	0.948
Patient’s interests first	Never/sometimes	Ref.	28.4	45.6	11.1				98.6	98.6	98.5			
	Usually/always		71.6	54.4	88.9	0.1	0.1–0.2	<0.001	1.4	1.4	1.5	0.5	0.2–0.9	0.028
			Seeking health information behavior for fetal development											
Internet search	Never/sometimes	Ref.	80.5	75.2	85.8				80.0	79.9	81.5			
	Usually/always		19.5	24.8	14.2	2.0	1.4–2.9	<0.001	20.0	20.1	18.5	1.1	0.6–2.1	0.749
Use of mobile app	Never/sometimes	Ref.	85.7	79.7	91.8				85.4	85.0	89.2			
	Usually/always		14.3	20.3	8.2	2.8	1.8–4.5	<0.001	14.6	15.0	10.8	1.4	0.6–3.3	0.359

ANC = antenatal care; PNC = postnatal care; Ref = reference group.

**Table 4:** The binary logistic regression analysis was conducted regarding patient-centered experiences and satisfaction with the hospital environment.

			ANC (*n* = 707)						PNC (*n* = 691)					
			Total (*n* = 707) %	Yes %	No %	OR	95% CI	*p*	Total (*n* = 691) %	Yes %	No %	OR	95% CI	*p*
Hospital cleanliness	Never/sometimes	Ref.	6.4	5.6	7.1				6.4	6.1	9.2			
	Usually/always		93.6	94.4	92.9	1.3	0.7–2.4	0.425	93.6	93.9	90.8	1.6	0.6–3.9	0.324
Safe and comfortable hospital environment	Never/sometimes	Ref.	5.1	3.7	6.5				5.2	5.4	3.1			
	Usually/always		94.9	96.3	93.5	1.8	0.9–3.7	0.087	94.8	94.6	96.9	0.6	0.1–2.4	0.423
Excellence of water quality	Unsatisfied	Ref.	20.1	11.5	28.7				20.1	18.1	40.0			
	Satisfied		79.9	88.5	71.3	3.1	2.1–4.6	<0.001	79.9	81.9	60.0	3.0	1.8–5.2	<0.001
Adequacy of drinking water	Unsatisfied	Ref.	91.9	92.4	91.5				91.9	91.7	93.8			
	Satisfied		8.1	7.6	8.5	0.9	0.5–1.5	0.654	8.1	8.3	6.2	1.4	0.5–3.9	0.547
Adequacy of water for handwashing	Unsatisfied	Ref.	8.2	9.9	6.5				8.4	7.3	18.5			
	Satisfied		91.8	90.1	93.5	6.4	0.4–1.1	0.110	91.6	92.7	81.5	2.9	1.4–5.7	0.003
Hospital laundry cleaning	Unsatisfied	Ref.	39.0	31.8	46.3				38.1	40.1	18.5			
	Satisfied		61.0	68.2	53.7	1.8	1.4–2.5	<0.001	61.9	59.9	81.5	0.3	0.2–0.6	0.001
Adequacy of water quality for medical service	Unsatisfied	Ref.	61.2	76.3	46.0				62.7	59.6	92.3			
	Satisfied		38.8	23.7	54.0	0.3	0.2–0.4	<0.001	37.3	40.4	7.7	8.1	3.2–20.5	<0.001

ANC = antenatal care; PNC = postnatal care; *p* < 0.05; Ref = reference group.

**Table 5:** Means and standard deviations of the patient-centered satisfaction score.

Patient-Centered Satisfaction Score	Total (*n* = 707)	ANC (*n* = 707)		U(p)	PNC (*n* = 691)		U(p)
		Yes (*n* = 355)	No (*n* = 352)		Yes (*n* = 626)	No (*n* = 65)	
Mean(SD)	8.4 (1.5)	8.4 (1.6)	8.5 (1.4)	611,066 (0.741)	8.4 (1.5)	8.9 (1.4)	16,072 (0.004)

Scores range from 0 to 10, where a higher score means higher satisfaction.

**Table 6:** Factors influencing maternal (patient) satisfaction scores.

	Model 1			Model 2		
	β	S.E	*p*	β	S.E	*p*
Age	−0.02	0.01	0.731	−0.01	0.01	0.771
Marriage period of 3 years or more	−0.06	0.14	0.154s	−0.05	0.13	0.231
More than elementary education	−0.07	0.12	0.082	−0.04	0.11	0.238
Patient-centered experiences with nurse				−0.01	0.03	0.915
Patient-centered experiences with midwife				0.11	0.04	0.021
Patient-centered experiences with doctor				−0.06	0.02	0.165
General care				−0.04	0.02	0.369
Satisfaction with hospital environment				0.25	0.05	<0.001
Satisfaction with hospital water quality				0.13	0.02	<0.001
Rights guaranteed				−0.04	0.05	0.304

## Data Availability

Any queries regarding the data used in this study can be directed toward the corresponding author. The dataset used in this study is available upon request.

## References

[1] World Bank (2023). Maternal Mortality Ratio. https://data.worldbank.org/indicator/SH.STA.MMRT?end=2020&locations=TZ&start=2000&view=chart.

[2] Cooke A., Mills T.A., Lavender T. (2010). ‘Informed and uninformed decision making’—Women’s reasoning, experiences and perceptions with regard to advanced maternal age and delayed childbearing: A meta-synthesis. Int. J. Nurs. Stud..

[3] Munguambe K., Boene H., Vidler M., Bique C., Sawchuck D., Firoz T., Makanga P.T., Qureshi R., Macete E., Menéndez C. (2016). Barriers and facilitators to health care seeking behaviours in pregnancy in rural communities of southern Mozambique. Reprod. Health.

[4] Nassoro M.M., Chiwanga E., Lilungulu A., Bintabara D. (2020). Maternal deaths due to obstetric haemorrhage in Dodoma Regional Referral Hospital, Tanzania. Obstet. Gynecol. Int..

[5] Bintabara D., Ernest A., Mpondo B. (2019). Health facility service availability and readiness to provide basic emergency obstetric and newborn care in a low-resource setting: Evidence from a Tanzania National Survey. BMJ Open.

[6] Wong E., Mavondo F., Fisher J. (2020). Patient feedback to improve quality of patient-centred care in public hospitals: A systematic review of the evidence. BMC Health Serv. Res..

[7] Miller P., Afulani P.A., Musange S., Sayingoza F., Walker D. (2021). Person-centered antenatal care and associated factors in Rwanda: A secondary analysis of program data. BMC Pregnancy Childbirth.

[8] Ng J.H., Luk B.H. (2019). Patient satisfaction: Concept analysis in the healthcare context. Patient Educ. Couns..

[9] Raposo M.L., Alves H.M., Duarte P.A. (2009). Dimensions of service quality and satisfaction in healthcare: A patient’s satisfaction index. Serv. Bus..

[10] Gupta S., Yamada G., Mpembeni R., Frumence G., Callaghan-Koru J.A., Stevenson R., Brandes N., Baqui A.H. (2014). Factors associated with four or more antenatal care visits and its decline among pregnant women in Tanzania between 1999 and 2010. PLoS ONE.

[11] Mutaganzwa C., Wibecan L., Iyer H.S., Nahimana E., Manzi A., Biziyaremye F., Nyishime M., Nkikabahizi F., Hirschhorn L.R., Magge H. (2018). Advancing the health of women and newborns: Predictors of patient satisfaction among women attending antenatal and maternity care in rural Rwanda. Int. J. Qual. Health Care.

[12] Wekesah F.M., Mbada C.E., Muula A.S., Kabiru C.W., Muthuri S.K., Izugbara C.O. (2016). Effective non-drug interventions for improving outcomes and quality of maternal health care in sub-Saharan Africa: A systematic review. Syst. Rev..

[13] Bergh K., Bishu S., Taddese H.B. (2022). Identifying the determinants of patient satisfaction in the context of antenatal care in Kenya, Tanzania, and Malawi using service provision assessment data. BMC Health Serv. Res..

[14] OECD (2022). Healthcare Quality Indicators. https://stats.oecd.org/Index.aspx?DataSetCode=HEALTH_HCQI.

[15] Centers for Medicare & Medicaid Services (2021). HCAHPS Quality Assurance Guidelines.

[16] UK National Health Service (2013). Imperial Survey Core Questionnaire. https://digital.nhs.uk/data-and-information/publications/statistical/health-survey-for-england.

[17] Yuill C., McCourt C., Cheyne H., Leister N. (2020). Women’s experiences of decision-making and informed choice about pregnancy and birth care: A systematic review and meta-synthesis of qualitative research. BMC Pregnancy Childbirth.

[18] Stapleton H., Kirkham M., Thomas G. (2002). Qualitative study of evidence based leaflets in maternity care. BMJ.

[19] Edwards A., Elwyn G., Mulley A. (2002). Explaining risks: Turning numerical data into meaningful pictures. BMJ.

[20] Thorne S.E., Paterson B.L. (2001). Health care professional support for self-care management in chronic illness: Insights from diabetes research. Patient Educ. Couns..

[21] Say R.E., Thomson R. (2003). The importance of patient preferences in treatment decisions—Challenges for doctors. BMJ.

[22] Hwang B.-D., Kim Y.-J. (2018). Evaluation of patient-centered healthcare provision in hospitals and general hospitals-based on patient experience assessment. Korean J. Health Serv. Manag..

[23] Ward J.K., Armitage G. (2012). Can patients report patient safety incidents in a hospital setting? A systematic review. BMJ Qual. Saf..

[24] Elias R.M., Fischer K.M., Siddiqui M.A., Coons T., Meyerhofer C.A., Pretzman H.J., Greig H.E., Stevens S.K., Burton M.C. (2021). A taxonomic review of patient complaints in adult hospital medicine. J. Patient Exp..

[25] Nowotny B.M., Loh E., Davies-Tuck M., Hodges R., Wallace E.M. (2019). Identifying quality improvement opportunities using patient complaints: Feasibility of using a complaints taxonomy in a metropolitan maternity service. J. Patient Saf. Risk Manag..

[26] Tanyi P.L., Pelser A. (2020). Towards an “age-friendly-hospital”: Older persons’ perceptions of an age-friendly hospital environment in Nigeria. Cogent Med..

[27] Adhikary G., Shawon M.S.R., Ali M.W., Shamsuzzaman M., Ahmed S., Shackelford K.A., Woldeab A., Alam N., Lim S.S., Levine A. (2018). Factors influencing patients’ satisfaction at different levels of health facilities in Bangladesh: Results from patient exit interviews. PLoS ONE.

[28] Nguyen T., Nguyen H., Dang A. (2020). Determinants of patient satisfaction: Lessons from large-scale inpatient interviews in Vietnam. PLoS ONE.

[29] Singh S., Kaur P., Rochwani R. (2013). Patient satisfaction levels in a tertiary care medical college hospital in Punjab, North India. Int. J. Res. Dev. Health.

[30] Srivastava A., Avan B.I., Rajbangshi P., Bhattacharyya S. (2015). Determinants of women’s satisfaction with maternal health care: A review of literature from developing countries. BMC Pregnancy Childbirth.

[31] Kebede D.B., Belachew Y.B., Selbana D.W., Gizaw A.B. (2020). Maternal satisfaction with antenatal care and associated factors among pregnant women in Hossana town. Int. J. Reprod. Med..

[32] Ghiasi A. (2021). Health information needs, sources of information, and barriers to accessing health information among pregnant women: A systematic review of research. J. Matern.-Fetal Neonatal Med..

[33] Javanmardi M., Noroozi M., Mostafavi F., Ashrafi-Rizi H. (2018). Internet usage among pregnant women for seeking health information: A review article. Iran. J. Nurs. Midwifery Res..

[34] Lagan B.M., Sinclair M., George Kernohan W. (2010). Internet use in pregnancy informs women’s decision making: A web-based survey. Birth.

[35] Lu Y., Zhang Z., Min K., Luo X., He Z. Pregnancy-related information seeking in online health communities: A qualitative study. Proceedings of the International Conference on Information.

[36] Nörby U., Noël-Cuppers B., Hristoskova S., Desai M., Härmark L., Steel M., El-Haddad C., Douarin L. (2021). Online information discrepancies regarding safety of medicine use during pregnancy and lactation: An IMI ConcePTION study. Expert Opin. Drug Saf..

[37] Lwoga E.T., Chigona W. (2017). Characteristics and factors that differentiate Internet users and non-users as information seekers: The case of rural women in Tanzania. Inf. Dev..

[38] Heri R., Yahya-Malima K.I., Malqvist M., Mselle L.T. (2023). Women’s Expectations of and Satisfaction with Antenatal Care Services in a Semi-Urban Setting in Tanzania and Associated Factors: A Cross-Sectional Survey. Healthcare.

